# Efficacy of a Hyaluronic Acid‐Based Gel Versus 0.20% Chlorhexidine in Nonsurgical Periodontal Therapy: A Single‐Blind Randomized Clinical Trial

**DOI:** 10.1111/odi.70345

**Published:** 2026-04-15

**Authors:** Andrea Scribante, Maurizio Pascadopoli, Matteo Pellegrini, Karyna Chekal, Andrea Butera

**Affiliations:** ^1^ Unit of Dental Hygiene, Section of Dentistry, Department of Clinical, Surgical, Diagnostic and Pediatric Sciences University of Pavia Pavia Italy; ^2^ Unit of Orthodontics and Pediatric Dentistry, Section of Dentistry, Department of Clinical, Surgical, Diagnostic and Pediatric Sciences University of Pavia Pavia Italy; ^3^ Section of Dentistry, Department of Clinical, Surgical, Diagnostic and Pediatric Sciences University of Pavia Pavia Italy

**Keywords:** chlorhexidine, dentistry, hyaluronic acid, nonsurgical periodontal therapy, periodontitis, randomized controlled trial

## Abstract

**Objective:**

To compare the adjunctive efficacy of a hyaluronic acid (HA) gel with 0.20% chlorhexidine (CHX) during nonsurgical periodontal therapy in periodontal patients over 6 months.

**Methods:**

A single‐blind, randomized, controlled trial included 40 healthy, nonsmoking adults with periodontitis. After supra‐ and subgingival debridement, patients used either HA gel (Aftoral; hyaluronic acid with glycerophosphoinositol and xylitol) or CHX mouthwash (Unidea; 0.20% chlorhexidine digluconate) once daily for 15 days at each time recall appointment. Clinical parameters—probing pocket depth (PPD, primary outcome), plaque control record (PCR), bleeding on probing (BoP), gingival recession (GR), tooth mobility (TM), clinical attachment level (CAL), and satisfaction (VAS)—were measured at baseline, 1, 3, and 6 months by a blinded examiner. Nonparametric tests were applied with significance at *p* < 0.05.

**Results:**

HA showed greater PPD reduction than CHX at 3 and 6 months (−0.44 mm vs. −0.26 mm; *p* < 0.01). PCR and BoP decreased more in the HA group (*p* < 0.05), while CAL gain was higher (−0.43 mm vs. −0.21 mm; *p* < 0.05). GR, TM, and VAS showed no differences. No adverse events occurred.

**Conclusions:**

Adjunctive HA gel improved PPD, PCR, BoP, and CAL compared with CHX, confirming safety, and clinical benefit.

## 1 Introduction

Periodontitis is a chronic inflammatory disease that affects the supporting tissues of the teeth, primarily caused by the accumulation of subgingival biofilm and dysbiosis in the oral microbiota (Kwon et al. 2021). In particular, periodontitis is characterized by progressive clinical attachment loss, increased probing depths, and alveolar bone resorption, often associated with pathogenic species from the “red complex,” including *Porphyromonas gingivalis* , *Treponema denticola* , and *Tannerella forsythia* (Socransky et al. 1998). The 2017 World Workshop classification provides a standardized framework for diagnosing and staging periodontitis, which aids in individualized treatment planning and risk assessment (Tonetti et al. 2018; Caton et al. 2018).

Nonsurgical periodontal therapy, including mechanical debridement and patient‐administered home care, remains the cornerstone of treatment (Kocher et al. 2025; Preda et al. 2021; Chan et al. 2025). Among chemical adjuncts, chlorhexidine (CHX) is considered the gold standard due to its broad‐spectrum antimicrobial activity and high substantivity (Eley 1999). Nevertheless, its long‐term use is limited by adverse effects such as tooth and mucosal staining, altered taste, and mucosal desquamation (Burns et al. 1997; Löe and Schiøtt 1970). Moreover, CHX does not selectively target pathogenic bacteria, and its repeated use can disrupt the ecological balance of the oral microbiome (Greenstein et al. 1986; Bartsch et al. 2025).

To address these limitations, alternative agents with antimicrobial and anti‐inflammatory properties but fewer side effects have been investigated (Bergamini et al. 2025; Dervisbegovic et al. 2025; Scribante et al. 2022; Minervini et al. 2024; Colombo et al. 2021; Gokul et al. 2025; Butera et al. 2024; Kardaras et al. 2024). Among natural adjuncts proposed as alternatives to chlorhexidine, essential oils and plant‐derived products have gained increasing attention due to their antimicrobial and anti‐inflammatory properties. Tea tree oil (*Melaleuca alternifolia*) has demonstrated antibacterial activity against oral pathogens and has been investigated as a mouthrinse in randomized clinical trials, showing improvements in plaque accumulation, bleeding indices, and probing depth without the staining and taste alterations commonly associated with chlorhexidine use (Ripari, Cera, et al. 2020). Similarly, coconut oil has been evaluated in the context of oil‐pulling therapy and has shown potential plaque‐reducing and anti‐inflammatory effects, possibly related to its lauric acid content and antimicrobial activity against oral microorganisms (Ripari, Filippone, et al. 2020). Although these natural agents have shown promising results, their clinical efficacy and long‐term performance remain variable and require further well‐designed randomized clinical trials.

Hyaluronic acid (HA), a naturally occurring glycosaminoglycan, has demonstrated bacteriostatic activity against periodontal pathogens, as well as tissue‐reparative, angiogenic, and anti‐inflammatory properties (Pirnazar et al. 1999; Dahiya and Kamal 2013). Clinical studies suggest that HA‐based formulations can reduce gingival inflammation and bleeding, and improve healing outcomes without the adverse effects typically associated with chlorhexidine (Jentsch et al. 2003; Mesa et al. 2002).

Despite these promising results, clinical evidence comparing the long‐term effectiveness of HA‐based gels to CHX in the management of periodontitis remains limited and inconclusive. Most available studies lack robust randomized designs, have short follow‐up periods, or assess a narrow range of clinical outcomes (Abdulkareem et al. 2020). For example, Ramanauskaite et al. (2024) reported significant microbiological improvements at 6 months when using cross‐linked hyaluronic acid as an adjunct, but failed to evaluate outcomes beyond this timeframe, limiting long‐term clinical inference. Similarly, the randomized controlled trial (RCT) by Ramanauskaite et al. (2023) confirmed superior short‐term clinical outcomes (up to 6 months) with the combination of CHX and HA, but did not assess sustainability beyond this period, nor did it isolate the individual contribution of HA. Moreover, Mamajiwala et al. (2021) extended their observation to 12 months, yet their design focused on HA as an adjunct to open flap debridement rather than nonsurgical therapy, and was limited by a small sample size and split‐mouth design. These methodological limitations underscore the need for well‐designed, long‐term RCT comparing HA‐based gels directly to CHX in nonsurgical periodontal treatment.

Therefore, the present single‐blind RCT aimed to compare the efficacy of a hyaluronic acid‐based oral gel containing xylitol and glycerophosphoinositol with a 0.20% chlorhexidine mouthwash as adjuncts to nonsurgical periodontal therapy in patients with periodontitis. Clinical parameters evaluated included Bleeding on Probing (BoP), Plaque Control Record (PCR), Probing Pocket Depth (PPD), Gingival Recession (GR), tooth mobility (TM), Clinical Attachment Level (CAL). In addition, overall product acceptance (taste, odor, consistency, persistence, and ease of application) was assessed using a Visual Analog Scale (VAS).

The first null hypothesis is that there is no significant difference in clinical periodontal parameters between the hyaluronic acid‐based gel and 0.20% chlorhexidine over a 6‐month follow‐up.

The second null hypothesis is that patient satisfaction does not differ significantly between the two treatment protocols.

## 2 Methods

### 2.1 Study Design

This was a monocentric, single‐blind, randomized, controlled, parallel‐group clinical trial conducted at the Dental Clinic of the University of Pavia between January 2023 and September 2023. The study protocol was approved by the Unit Internal Review Board (approval number: 2022–0302). The trial was registered on ClinicalTrials.gov (NCT number: NCT05312606).

The study was conducted in accordance with the Declaration of Helsinki on experimentation involving human subjects, and the principles of Good Clinical Practice. The manuscript was prepared in accordance with the CONSORT 2025 checklist for RCT (Hopewell et al. 2025) (Table S1). Written informed consent was obtained from all participants prior to their enrollment in the study.

### 2.2 Participants

All participant recruitment, clinical assessments, and data collection were conducted at the Dental Clinic of the Department of Clinical, Surgical, Diagnostic and Pediatric Sciences, University of Pavia, 27,100 Pavia, Italy. Patients were consecutively selected among those referred for nonsurgical periodontal therapy and diagnosed with periodontitis according to the 2017 World Workshop classification system (Tonetti et al. 2018).

### 2.3 Eligibility Criteria for Participants

To ensure the homogeneity of the study population, specific inclusion and exclusion criteria were applied. Eligible participants were adults aged 18 years or older, in good general health, diagnosed with periodontitis according to the 2017 World Workshop classification (Tonetti et al. 2018). Specifically, inclusion required the presence of interdental clinical attachment loss of 3–4 mm at two or more nonadjacent sites, probing pocket depth not exceeding 5 mm, and radiographic evidence of alveolar bone loss confined to the coronal third of the root (15%–33%), with no history of tooth loss attributable to periodontitis. Only patients with absence of tooth mobility (Miller class 0) were considered eligible. In addition, participants had to present at least two periodontal sites with PPD ≥ 4 mm and bleeding on probing at baseline, no previous periodontal therapy within the 6 months prior to enrollment, adequate compliance with oral hygiene instructions, and willingness to attend all scheduled follow‐up visits.

Exclusion criteria comprised current smoking or the use of tobacco products, pregnancy or breastfeeding, and the presence of systemic conditions known to affect periodontal health, such as uncontrolled diabetes mellitus or immunodeficiencies. Additional exclusion criteria included antibiotic or anti‐inflammatory therapy within the past 3 months, known allergies to any ingredients of the study products, poor oral hygiene compliance, or inability to adhere to the clinical protocol.

### 2.4 Clinical Procedure and Outcomes

All clinical procedures were carried out in accordance with the inclusion and exclusion criteria described above. At baseline (T0), a trained and calibrated operator, blinded to group allocation, used a calibrated periodontal probe (UNC probe 15; Hu‐Friedy, Chicago, IL, USA) to evaluate patients and to record the following parameters at both patient‐level and tooth‐level, on six sites per tooth (mesiobuccal, buccal, distobuccal, mesiolingual, lingual, distolingual): BoP (Doornewaard et al. 2018), PPD (AbdulAzeez and Alkinani 2021), PCR (O'Leary et al. 1972), GR (Cairo et al. 2011), TM (Miller 1950), and CAL (Needleman et al. 2018). Overall product acceptance was assessed using VAS (Thong et al. 2018).

The primary outcome of the study was the change in PPD from baseline (T0) to the follow‐up time points, assessed at both tooth‐level and patient‐level. Secondary outcomes included changes in BoP, PCR, GR, TM, CAL, and VAS across the same time points.

For the analysis at the patient‐level, mean values for PPD, GR, and CAL were calculated by averaging all measurements collected across all teeth. TM at the patient‐level was calculated as the mean of all mobility scores recorded for these teeth. Conversely, BoP and PCR were expressed as percentages, representing the proportion of periodontal sites exhibiting detectable bleeding or visible plaque relative to the total number of examined sites per patient.

At the tooth‐level, all clinical parameters were recorded at six sites per tooth (mesiobuccal, buccal, distobuccal, mesiolingual, lingual, distolingual). PPD, GR, and CAL were documented in millimeters per site, while BoP and PCR were scored dichotomously (presence/absence) at each surface. TM was assessed per individual tooth using the Miller classification system.

Clinical assessments were performed at four time points: T0 (baseline), T1 (1 month), T2 (3 months), and T3 (6 months).

Before initiating the clinical phase, examiner calibration was performed on a group of 10 patients with periodontitis, each presenting at least one site with PPD ≥ 4 mm. Double measurements were taken 48 h apart. Calibration was deemed acceptable if ≥ 90% of repeated measurements showed a deviation ≤ 1 mm, ensuring intra‐examiner consistency (Mariani et al. 2020).

Following baseline assessment, nonsurgical periodontal debridement was performed, consisting of supragingival scaling with an ultrasonic device (Cavitron Select SPS, Dentsply Sirona, USA), and subgingival instrumentation with Gracey curettes (Hu‐Friedy, Chicago, IL, USA) in sites with PPD ≥ 4 mm and/or BoP. All patients also received air polishing with glycine powder (Air‐Flow, EMS, Nyon, Switzerland) to facilitate biofilm disruption.

Participants were randomly assigned to one of two parallel groups. The test group received domiciliary application of a hyaluronic acid‐based gel (Aftoral Oral Gel, Healmann Health Care, Milan, Italy), containing glycerophosphoinositol, lysine salt, hyaluronic acid, and sodium salt. The gel was applied once daily for 15 days in undiluted form, using 10 mL in total. The control group used a mouthwash containing 0.20% chlorhexidine digluconate (Unidea Mouthwash, Unifarco S.p.A., Santa Giustina, Italy), also applied once daily for 15 days.

At T1 and T2, sites exhibiting signs of persistent inflammation (BoP and/or PPD ≥ 5 mm) received additional subgingival decontamination using glycine powder and the PerioPolishing System according to a predefined standardized protocol, applied uniformly across all groups. After T1 and T2 reevaluation, the adjunctive gel/mouthwash was applied for 15 days as well. At T3, all patients received a complete nonsurgical periodontal session, including ultrasonic scaling, manual instrumentation with Gracey curettes where indicated, and polishing with glycine powder.

Patients received standardized verbal and written instructions on how to apply the assigned product. All procedures were repeated consistently by the same trained operator throughout the study to ensure reliability.

The composition of the two tested mouthwashes is detailed in Table 1.

**TABLE 1:** Composition of the tested products.

Product	Manufacturer	Composition
Aftoral oral gel	Healmann Health Care S.r.l. (Milan, Italy)	Glycerophosphoinositol, Lysine salt, Hyaluronic acid, Sodium salt
Unidea mouthwash	Unifarco S.p.A. (Santa Giustina, Italy)	Aqua, Glycerin, Propylene glycol, PEG‐40 hydrogenated castor oil, Aroma, Methylparaben, Potassium acesulfame, Propylparaben, Sodium fluoride (226 ppm F)

### 2.5 Sample Size Calculation

The sample size calculation (alpha = 0.05; power = 90%) for two independent study groups and a continuous primary endpoint was calculated.

The following mathematical formula was used for the sample size calculation:

$$\text{Sample size}=\frac{\left(\sigma_{2}^{1}+\sigma_{2}^{2}\right)\left(Z_{\left(1-\frac{\alpha }{2}\right)}+Z_{\left(1-\beta \right)}\right)}{\Delta^{2}}$$

In the sample size calculation, *Z* represents the value from the standard normal distribution corresponding to a 5% alpha level (1.96), *σ*_1_ and *σ*_2_ denote the variance of the two means, *Z* is the critical *Z* value for a given α error and *β* error, while Δ is the absolute difference between the two means. PPD was selected as the primary outcome variable. Based on previous data, a mean value of 4.40 mm was anticipated in the test group, with an expected difference between groups of 0.71 mm and a standard deviation of 0.70 mm (Bertl et al. 2024). Accordingly, a total of 20 patients per group (40 in total) was calculated to be necessary to ensure adequate statistical power (90%) within the parallel‐group design.

### 2.6 Randomization

Using a block randomization procedure, the data analyst generated a randomization sequence with permuted blocks in R software (version 4.5.1; R Foundation for Statistical Computing, Vienna, Austria) using the blockrand package. A total of 40 participants were randomly assigned in a 1:1 ratio to either the test or control group. Allocation was concealed by means of the computer‐generated randomization list in R software, which was accessed only by the care provider at the time of assignment.

Participants in the test group received nonsurgical periodontal therapy combined with domiciliary use of Aftoral Oral Gel (Healmann Health Care, Milan, Italy), containing hyaluronic acid, glycerophosphoinositol, and xylitol, applied once daily for 15 days.

Participants in the control group received the same periodontal therapy along with Unidea Mouthwash (Unifarco S.p.A., Santa Giustina, Italy), containing 0.20% chlorhexidine digluconate, also used once daily for 15 days. Participants were instructed to follow the assigned regimen precisely, and no additional domiciliary treatments were introduced.

### 2.7 Blinding and Calibration

For the domiciliary protocols, the two products (Aftoral Oral Gel and Unidea Mouthwash) were supplied in their original, clearly labeled retail packaging, allowing participants to follow the assigned regimen correctly. Due to the visible differences in formulation and mode of use, blinding of participants was not feasible. Group allocation and product instructions were managed by a second operator (care provider), who was not involved in clinical measurements. Importantly, the trained clinical examiner responsible for periodontal assessments (PPD, PCR, BoP, GR, TM, and CAL) was fully blinded to group assignment throughout the study. This was achieved by ensuring that the care provider delivered the product and instructions to participants outside the presence of the examiner. In addition, the statistician performing data analysis was blinded to treatment allocation and outcome values during the entire statistical processing phase.

Prior to the start of the study, the examiner underwent a calibration process involving 10 patients with periodontitis, each presenting at least one site with a probing pocket depth ≥ 4 mm. Periodontal parameters were recorded on two separate occasions, 48 h apart. Calibration was considered successful when at least 90% of repeated measurements showed a discrepancy of less than 1 mm, thereby ensuring a high level of intra‐examiner reliability and methodological consistency (Mariani et al. 2020).

### 2.8 Statistical Analysis

Statistical analyses were performed using R software (version 4.5.1; R Foundation for Statistical Computing, Vienna, Austria). Data were evaluated both at the tooth‐level and patient‐level. Descriptive statistics, including means, standard deviations, medians, ranges, 95% confidence intervals, were calculated for each clinical parameter within the two study groups.

The normality of data distribution was assessed using the Kolmogorov–Smirnov test. Since most variables did not follow a normal distribution, Nonparametric tests were applied. Between‐group comparisons were conducted using the Kruskal–Wallis test, followed by Dunn's post hoc test for pairwise multiple comparisons. For within‐group comparisons over time, the Friedman test for repeated measures was used, also followed by Dunn's post hoc analysis to identify specific time points with significant differences. Tooth‐level data were analyzed with Nonparametric Aligned Rank Transform ANOVA followed by Dunn's test as post hoc. A letter‐based grouping method was used to facilitate the interpretation of statistically significant differences across comparisons (Piepho 2004).

The level of statistical significance was set at *p* < 0.05 for all tests.

## 3 Results

### 3.1 Participants and Recruitment

Figure 1 shows the CONSORT 2025 flow diagram of the study (Hopewell et al. 2025). After screening, 40 patients fulfilling the inclusion criteria were recruited at the Unit of Dental Hygiene, Section of Dentistry, Department of Clinical, Surgical, Diagnostic and Pediatric Sciences, University of Pavia, Italy. Recruitment began in January 2023 and was completed in September 2023.

**FIGURE 1:**
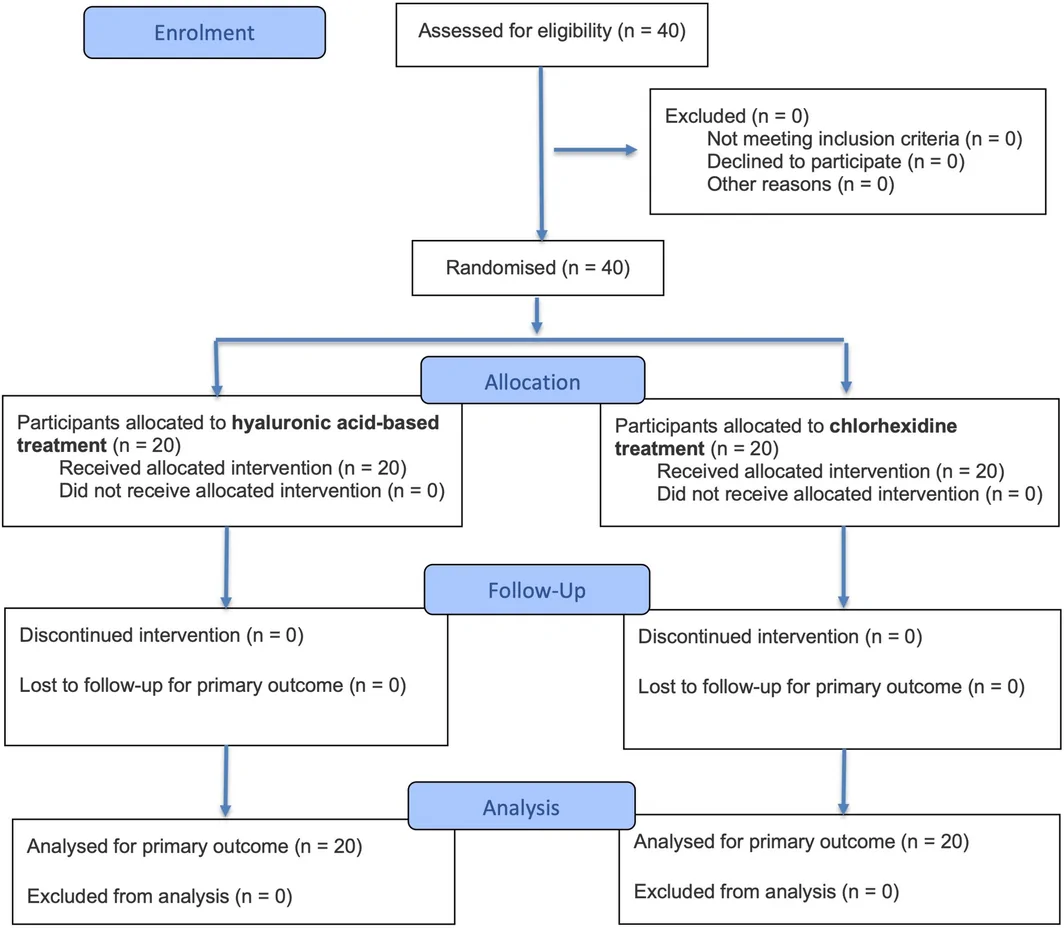
CONSORT 2025 flow diagram.

Participants were randomly allocated in a 1:1 ratio into two parallel groups: 20 patients were assigned to the test group (Aftoral Oral Gel), and 20 to the control group (Unidea Mouthwash).

All participants completed the scheduled follow‐up visits at 1 (T1), 3 (T2), and 6 months (T3). No patients discontinued the intervention or were lost to follow‐up. Importantly, no adverse events or harms related to the use of either mouthwash were reported during the study period.

### 3.2 Demographic Characteristics

The demographic characteristics of the study sample are presented in Table 2. The study population consisted of 18 male (45%) and 22 female (55%) participants. The overall mean age was 60.43 ± 10.78 years, with a range of 34–86 years. The mean age of male participants was 61.56 ± 12.68 years (range: 36–86), while that of female participants was 59.51 ± 9.14 years (range: 34–74). Participants in the control group had a mean age of 61.77 ± 13.94 years (range: 36–76), whereas those in the trial group had a mean age of 61.38 ± 10.08 years (range: 43–86). None of the participants were current smokers, and no systemic conditions or medications known to affect periodontal health were reported, thereby minimizing potential confounding factors.

**TABLE 2:** Baseline demographic and dental characteristics of the study population.

Variable	Value
*Age* (*years*)	
Overall (mean ± SD, range)	60.43 ± 10.78 (34–86)
Male (mean ± SD, range)	61.56 ± 12.68 (36–86)
Female (mean ± SD, range)	59.51 ± 9.14 (34–74)
Control group (mean ± SD, range)	61.77 ± 13.94 (36–76)
Trial group (mean ± SD, range)	61.38 ± 10.08 (43–86)
*Gender distribution* (*n* (*%*))	
Male	18 (45.00)
Female	22 (55.00)
*Teeth position* (*n* (*%*))—*Control group*	
Maxillary	78 (70.27)
Mandibular	33 (29.73)
Quadrant 1	38 (34.23)
Quadrant 2	40 (36.04)
Quadrant 3	16 (14.41)
Quadrant 4	17 (15.32)
*Teeth position* (*n* (*%*))—*Test group*	
Maxillary	81 (72.97)
Mandibular	30 (27.03)
Quadrant 1	42 (37.84)
Quadrant 2	39 (35.13)
Quadrant 3	9 (8.11)
Quadrant 4	21 (18.92)

Abbreviation: SD, standard deviation.

### 3.3 Outcomes

In the control group, the mean PPD at the tooth‐level showed a slight reduction from 3.95 ± 1.84 mm (95% CI: 3.09–4.81) at baseline (T0) to 3.69 ± 1.50 mm (95% CI: 2.98–4.39) at 6 months (T3). In the test group, the mean PPD decreased more consistently, from 3.93 ± 1.57 mm (95% CI: 3.19–4.66) at T0 to 3.49 ± 1.04 mm (95% CI: 3.01–3.98) at T3. According to the intragroup analysis, in the control group T0 values were not significantly different from T1 and T2, while a significant reduction was observed at T3. In the test group, T0 and T1 were not significantly different, whereas T2 and T3 showed significantly lower values compared with baseline. Intergroup comparisons at each time point revealed no statistically significant differences between the test and control groups. At the patient‐level, the mean PPD in the control group decreased from 3.94 ± 0.45 mm (95% CI: 3.73–4.15) at T0 to 3.68 ± 0.42 mm (95% CI: 3.48–3.87) at T3. In the test group, values decreased from 3.96 ± 0.53 mm (95% CI: 3.71–4.21) at T0 to 3.51 ± 0.38 mm (95% CI: 3.33–3.69) at T3. Intragroup analysis showed that, in the control group, T3 was significantly lower than T0, while T1 and T2 did not differ significantly from baseline. In the test group, T2 and T3 were significantly lower than T0, while T1 showed no significant difference. Similarly to the tooth‐level analysis, no statistically significant intergroup differences were observed at any time point. Comprehensive descriptive data and statistical comparisons are reported in Table 3.

**TABLE 3:** Descriptive statistics and intragroup/intergroup comparisons of PPD tooth‐level and patient‐level values across all time points (T0–T3) in the Test and Control groups.

Group	Time	Mean ± SD	Range	Median	CI (95%)	Intragroup (T0–T3)^a^	Intergroup (control‐test)^a^
Tooth‐level							
Control (*n* = 20)	T0	3.95 ± 1.84	3.00–15.00	3.00	[3.09–4.81]	a	A
	T1	3.80 ± 1.63	3.00–15.00	3.00	[3.03–4.56]	a,b	A
	T2	3.77 ± 1.61	3.00–15.00	3.00	[3.02–4.52]	a,b	A
	T3	3.69 ± 1.50	3.00–15.00	3.00	[2.98–4.39]	b	A
Test (*n* = 20)	T0	3.93 ± 1.57	3.00–15.00	3.00	[3.19–4.66]	a	A
	T1	3.66 ± 1.27	3.00–15.00	3.00	[3.07–4.26]	a,b	A
	T2	3.55 ± 1.14	3.00–14.00	3.00	[3.01–4.08]	b	A
	T3	3.49 ± 1.04	3.00–12.00	3.00	[3.01–3.98]	b	A

Patient‐level							
Control (*n* = 20)	T0	3.94 ± 0.45	3.40–5.06	3.84	[3.73–4.15]	a	A
	T1	3.78 ± 0.44	3.27–4.94	3.74	[3.57–3.99]	a,b	A
	T2	3.76 ± 0.44	3.25–4.92	3.69	[3.56–3.96]	a	A
	T3	3.68 ± 0.42	3.21–4.75	3.58	[3.48–3.87]	b	A
Test (*n* = 20)	T0	3.96 ± 0.53	3.46–5.79	3.86	[3.71–4.21]	a	A
	T1	3.69 ± 0.47	3.29–5.46	3.61	[3.47–3.91]	a,b	A
	T2	3.57 ± 0.47	3.17–5.29	3.47	[3.35–3.79]	b	A
	T3	3.51 ± 0.38	3.13–4.83	3.40	[3.33–3.69]	b	A

Abbreviations: max, maximum; min, minimum; SD, standard deviation; T0, baseline; T1, 1 month; T2, 3 months; T3, 6 months.

^a^ Means presenting at least one identical letter are not significantly different (*p* > 0.05). Intragroup analysis considered all the time frames of the study for the comparisons (lowercase letters), while intergroup analysis considered only Control vs. Test comparison at each time frame (uppercase letters).

Regarding PCR, tooth‐level values at baseline were comparable between groups, with a mean of 0.75 ± 0.43 (95% CI: 0.72–0.79) in the control group and 0.82 ± 0.39 (95% CI: 0.63–1.00) in the test group. In the control group, values decreased to 0.49 ± 0.50 (95% CI: 0.26–0.73) at T3, while in the test group they decreased to 0.46 ± 0.50 (95% CI: 0.23–0.70). Intragroup analysis showed that, in both groups, PCR values at all follow‐up time points (T1, T2, and T3) were significantly lower than baseline, while no significant differences were observed among the follow‐up time points themselves. Intergroup comparisons revealed no statistically significant differences between the test and control groups at any time point. At the patient‐level, the mean PCR decreased from 75.55% ± 24.75% (95% CI: 63.97–87.13) at T0 to 54.00% ± 30.02% (95% CI: 39.95–68.05) at T3 in the control group, and from 80.75% ± 24.38% (95% CI: 69.34–92.16) at T0 to 51.05% ± 20.59% (95% CI: 41.41–60.69) at T3 in the test group. Intragroup analysis indicated that, in the control group, no statistically significant changes were observed over time. In contrast, in the test group, a statistically significant reduction was observed only at T3 compared with baseline, while intermediate time points did not differ significantly. Similarly to the tooth‐level analysis, no statistically significant intergroup differences were found at any time point. Detailed descriptive statistics and intra‐ and intergroup comparisons are presented in Table 4.

**TABLE 4:** Descriptive statistics and intragroup/intergroup comparisons of PCR tooth‐level and patient‐level values across all time points (T0–T3) in the Test and Control groups.

Group	Time	Mean	Range	Median	CI (95%)	Intragroup (T0–T3)^a^	Intergroup (control‐test)^a^
Tooth‐level							
Control (*n* = 20)	T0	0.75 ± 0.43	0.00–1.00	1.00	[0.72–0.79]	a	A
	T1	0.58 ± 0.49	0.00–1.00	1.00	[0.35–0.81]	b	A
	T2	0.53 ± 0.50	0.00–1.00	1.00	[0.30–0.77]	b	A
	T3	0.49 ± 0.50	0.00–1.00	0.00	[0.26–0.73]	b	A
Test (*n* = 20)	T0	0.82 ± 0.39	0.00–1.00	1.00	[0.63–1.00]	a	A
	T1	0.58 ± 0.49	0.00–1.00	1.00	[0.35–0.81]	b	A
	T2	0.54 ± 0.50	0.00–1.00	1.00	[0.31–0.77]	b	A
	T3	0.46 ± 0.50	0.00–1.00	0.00	[0.23–0.70]	b	A

Patient‐level							
Control (*n* = 20)	T0	75.55 ± 24.75	20.00–100.00	71.50	[63.97–87.13]	a	A
	T1	60.87 ± 21.19	17.00–100.000	60.00	[50.95–70.78]	a	A
	T2	56.90 ± 22.08	11.00–85.00	54.50	[46.57–67.23]	a	A
	T3	54.00 ± 30.02	12.00–100.00	50.00	[39.95–68.05]	a	A
Test (*n* = 20)	T0	80.75 ± 24.38	32.00–100.00	95.50	[69.34–92.16]	a	A
	T1	62.36 ± 19.79	30.00–100.00	67.00	[53.10–71.62]	a,b	A
	T2	56.35 ± 20.79	20.00–100.00	57.00	[46.62–66.08]	a,b	A
	T3	51.05 ± 20.59	15.00–100.00	55.00	[41.41–60.69]	b	A

Abbreviations: max, maximum; min, minimum; SD, standard deviation; T0, baseline; T1, 1 month; T2, 3 months; T3, 6 months.

^a^ Means presenting at least one identical letter are not significantly different (*p* > 0.05). Intragroup analysis considered all the time frames of the study for the comparisons (lowercase letters), while intergroup analysis considered only Control vs. Test comparison at each time frame (uppercase letters).

For BoP, baseline tooth‐level values were 0.39 ± 0.49 (95% CI: 0.35–0.43) in the control group and 0.28 ± 0.45 (95% CI: 0.07–0.49) in the test group. At T3, mean values were 0.29 ± 0.45 (95% CI: 0.08–0.50) in the control group and 0.15 ± 0.36 (95% CI: −0.02–0.32) in the test group. Intragroup analysis showed no statistically significant changes over time in either group at the tooth‐level. Intergroup comparisons revealed significantly lower BoP values in the test group compared with the control group at T1 and T3, while no significant differences were observed at T0 and T2. At the patient‐level, mean BoP in the control group decreased from 31.16% ± 26.86% (95% CI: 18.59–43.73) at T0 to 21.65% ± 22.01% (95% CI: 11.35–31.95) at T3. In the test group, values decreased more consistently, from 27.16% ± 20.77% (95% CI: 17.44–36.88) at T0 to 12.45% ± 9.56% (95% CI: 7.98–16.92) at T3. Intragroup analysis indicated that no statistically significant changes were observed in the control group over time. In contrast, in the test group, a statistically significant reduction was observed at T3 compared with baseline, while intermediate time points did not differ significantly. Intergroup comparisons at the patient‐level showed no statistically significant differences between groups at any time point. Detailed descriptive data and statistical comparisons are provided in Table 5.

**TABLE 5:** Descriptive statistics and intragroup/intergroup comparisons of BoP tooth‐level and patient‐level values across all time points (T0–T3) in the Test and Control groups.

Group	Time	Mean	Range	Median	CI 95%	Intragroup (T0–T3)^a^	Intergroup (control‐test)^a^
Tooth‐level							
Control (*n* = 20)	T0	0.39 ± 0.49	0.00–1.00	0.00	[0.35–0.43]	a	A
	T1	0.38 ± 0.48	0.00–1.00	0.00	[0.15–0.60]	a	A
	T2	0.27 ± 0.45	0.00–1.00	0.00	[0.07–0.48]	a	A
	T3	0.29 ± 0.45	0.00–1.00	0.00	[0.08–0.50]	a	A
Test (*n* = 20)	T0	0.28 ± 0.45	0.00–1.00	0.00	[0.07–0.49]	a	A
	T1	0.20 ± 0.40	0.00–1.00	0.00	[0.01–0.39]	a	B
	T2	0.18 ± 0.38	0.00–1.00	0.00	[0.00–0.35]	a	A
	T3	0.15 ± 0.36	0.00–1.00	0.00	[−0.02–0.32]	a	B

Patient‐level							
Control (*n* = 20)	T0	31.16 ± 26.86	2.70–100.00	23.06	[18.59–43.73]	a	A
	T1	26.24 ± 20.93	3.40–78.57	19.55	[16.44–36.03]	a	A
	T2	20.46 ± 19.44	3.40–71.42	15.00	[11.36–29.56]	a	A
	T3	21.65 ± 22.01	4.00–100.00	15.65	[11.35–31.95]	a	A
Test (*n* = 20)	T0	27.16 ± 20.77	4.00–79.40	21.40	[17.44–36.88]	a	A
	T1	21.07 ± 16.31	2.77–19.35	19.35	[13.44–28.71]	a,b	A
	T2	16.45 ± 12.91	5.00–60.00	12.75	[10.41–22.49]	a,b	A
	T3	12.45 ± 9.56	2.30–34.10	10.10	[7.98–16.92]	b	A

Abbreviations: max, maximum; min, minimum; SD, standard deviation; T0, baseline; T1, 1 month; T2, 3 months; T3, 6 months.

^a^ Means presenting at least one identical letter are not significantly different (*p* > 0.05). Intragroup analysis considered all the time frames of the study for the comparisons (lowercase letters), while intergroup analysis considered only Control vs. Test comparison at each time frame (uppercase letters).

When GR was considered, tooth‐level means in the control group remained stable throughout the study, with values of 0.52 ± 1.40 mm (95% CI: 0.41–0.62) at baseline (T0) and 0.59 ± 1.55 mm (95% CI: −0.13–1.31) at T3. In the test group, GR values were consistently lower and remained unchanged over time, with a mean of 0.23 ± 0.90 mm (95% CI: −0.19–0.66) at both T0 and T3. Intragroup analysis showed no statistically significant changes over time in either group. Intergroup comparisons also revealed no statistically significant differences between the test and control groups at any time point. At the patient‐level, the control group presented a mean GR of 0.52 ± 0.88 mm (95% CI: 0.10–0.93) at baseline, which slightly increased to 0.58 ± 0.92 mm (95% CI: 0.16–1.01) at T3. The test group maintained stable values across all time points, with a mean of 0.23 ± 0.45 mm (95% CI: 0.03–0.44) throughout the study. Similarly, intragroup analysis showed no statistically significant changes over time in either group, and no statistically significant intergroup differences were observed at any time point. Descriptive and comparative data are summarized in Table 6.

**TABLE 6:** Descriptive statistics and intragroup/intergroup comparisons of GR tooth‐level and patient‐level values across all time points (T0–T3) in the Test and Control groups.

Group	Time	Mean	Range	Median	CI 95%	Intragroup (T0–T3)^a^	Intergroup (control‐test)^a^
Tooth‐level							
Control (*n* = 20)	T0	0.52 ± 1.40	0.00–9.00	0.00	[0.41–0.62]	a	A
	T1	0.55 ± 1.48	0.00–9.00	0.00	[−0.14–1.25]	a	A
	T2	0.56 ± 1.51	0.00–10.00	0.00	[−0.41–1.27]	a	A
	T3	0.59 ± 1.55	0.00–10.00	0.00	[−0.13–1.31]	a	A
Test (*n* = 20)	T0	0.23 ± 0.90	0.00–6.00	0.00	[−0.19–0.66]	a	A
	T1	0.23 ± 0.90	0.00–6.00	0.00	[−0.19–0.66]	a	A
	T2	0.23 ± 0.90	0.00–6.00	0.00	[−0.19–0.66]	a	A
	T3	0.23 ± 0.90	0.00–6.00	0.00	[−0.19–0.66]	a	A

Patient‐level							
Control (*n* = 20)	T0	0.52 ± 0.88	0.00–3.75	0.19	[0.10–0.93]	a	A
	T1	0.55 ± 0.91	0.00–3.92	0.23	[0.12–0.98]	a	A
	T2	0.56 ± 0.91	0.00–3.92	0.23	[0.13–0.99]	a	A
	T3	0.58 ± 0.92	0.00–3.92	0.23	[0.16–1.01]	a	A
Test (*n* = 20)	T0	0.23 ± 0.45	0.00–1.90	0.04	[0.03–0.44]	a	A
	T1	0.23 ± 0.45	0.00–1.90	0.04	[0.03–0.44]	a	A
	T2	0.23 ± 0.45	0.00–1.90	0.04	[0.03–0.44]	a	A
	T3	0.23 ± 00.45	0.00–1.90	0.04	[0.03–0.44]	a	A

Abbreviations: max, maximum; min, minimum; SD, standard deviation; T0, baseline; T1, 1 month; T2, 3 months; T3, 6 months.

^a^ Means presenting at least one identical letter are not significantly different (*p* > 0.05). Intragroup analysis considered all the time frames of the study for the comparisons (lowercase letters), while intergroup analysis considered only Control vs. Test comparison at each time frame (uppercase letters).

With regard to TM, tooth‐level values remained stable over time in both groups. In the control group, mean TM was 0.10 ± 0.43 (95% CI: −0.10–0.13) at baseline (T0) and remained unchanged at all subsequent time points (T1–T3). Similarly, in the test group, mean TM was 0.09 ± 0.42 (95% CI: −0.10–0.29) at T0 and showed no variation throughout the follow‐up period. Intragroup analysis showed no statistically significant changes over time in either group. Intergroup comparisons also revealed no statistically significant differences between the test and control groups at any time point. At the patient‐level, the control group showed mean TM values of 0.10 ± 0.22 (95% CI: 0.00–0.21) at baseline, which remained unchanged through T3. The test group presented a mean TM of 0.09 ± 0.28 (95% CI: −0.04–0.22) at T0, with no variations across the follow‐up period. Similarly, no statistically significant intragroup changes or intergroup differences were observed at the patient‐level. Detailed descriptive and comparative results are presented in Table 7.

**TABLE 7:** Descriptive statistics and intragroup/intergroup comparisons of TM tooth‐level and patient‐level values across all time points (T0–T3) in the Test and Control groups.

Group	Time	Mean	Range	Median	CI 95%	Intragroup (T0–T3)^a^	Intergroup (control‐test)^a^
Tooth‐level							
Control (*n* = 20)	T0	0.10 ± 0.43	0.00–2‐00	0.00	[−0.10–0.13]	a	A
	T1	0.10 ± 0.43	0.00–2‐00	0.00	[−0.10–0.13]	a	A
	T2	0.10 ± 0.43	0.00–2‐00	0.00	[−0.10–0.13]	a	A
	T3	0.10 ± 0.43	0.00–2‐00	0.00	[−0.10–0.13]	a	A
Test (*n* = 20)	T0	0.09 ± 0.42	0.00–2‐00	0.00	[−0.10–0.29]	a	A
	T1	0.09 ± 0.42	0.00–2‐00	0.00	[−0.10–0.29]	a	A
	T2	0.09 ± 0.42	0.00–2‐00	0.00	[−0.10–0.29]	a	A
	T3	0.09 ± 0.42	0.00–2‐00	0.00	[−0.10–0.29]	a	A

Patient‐level							
Control (*n* = 20)	T0	0.10 ± 0.22	0.10–0.10	0.22	[0.00–0.21]	a	A
	T1	0.10 ± 0.22	0.10–0.10	0.22	[0.00–0.21]	a	A
	T2	0.10 ± 0.22	0.10–0.10	0.22	[0.00–0.21]	a	A
	T3	0.10 ± 0.22	0.10–0.10	0.22	[0.00–0.21]	a	A
Test (*n* = 20)	T0	0.09 ± 0.28	0.00–1.00	0.00	[−0.04–0.22]	a	A
	T1	0.09 ± 0.28	0.00–1.00	0.00	[−0.04–0.22]	a	A
	T2	0.09 ± 0.28	0.00–1.00	0.00	[−0.04–0.22]	a	A
	T3	0.09 ± 0.28	0.00–1.00	0.00	[−0.04–0.22]	a	A

Abbreviations: max, maximum; min, minimum; SD, standard deviation; T0, baseline; T1, 1 month; T2, 3 months; T3, 6 months.

^a^ Means presenting at least one identical letter are not significantly different (*p* > 0.05). Intragroup analysis considered all the time frames of the study for the comparisons (lowercase letters), while intergroup analysis considered only Control vs. Test comparison at each time frame (uppercase letters).

Looking at CAL, tooth‐level values in the control group showed minimal changes across time points, with a mean of 4.47 ± 2.39 mm (95% CI: 4.29–4.66) at baseline (T0), slightly decreasing to 4.28 ± 2.23 mm (95% CI: 3.23–5.32) at T3. In the test group, a greater reduction was observed, from 4.16 ± 1.84 mm (95% CI: 3.30–5.02) at T0 to 3.73 ± 1.39 mm (95% CI: 3.08–4.37) at T3. Intragroup analysis showed no statistically significant changes over time in the control group. In contrast, in the test group, CAL values at T2 and T3 were significantly lower than baseline, while T1 did not differ significantly. At the patient‐level, the control group showed a mean CAL of 4.46 ± 1.05 mm (95% CI: 3.97–4.95) at T0 and 4.26 ± 1.08 mm (95% CI: 3.76–4.77) at T3. In the test group, values decreased from 4.19 ± 0.73 mm (95% CI: 3.85–4.54) at T0 to 3.74 ± 0.60 mm (95% CI: 3.46–4.02) at T3. Intragroup analysis confirmed that, in the control group, a statistically significant reduction was observed only at T3 compared with baseline, whereas in the test group significant reductions were already evident at T2 and maintained at T3. Intergroup comparisons revealed no statistically significant differences between the test and control groups at any time point, both at the tooth‐level and patient‐level. A comprehensive overview of CAL descriptive statistics and intragroup/intergroup comparisons is provided in Table 8.

**TABLE 8:** Descriptive statistics and intragroup/intergroup comparisons of CAL tooth‐level and patient‐level values across all time points (T0–T3) in the Test and Control groups.

Group	Time	Mean	Range	Median	CI 95%	Intragroup (T0–T3)^a^	Intergroup (control‐test)^a^
Tooth‐level							
Control (*n* = 20)	T0	4.47 ± 2.39	3.00–15.00	3.00	[4.29–4.66]	a	A
	T1	4.35 ± 2.30	3.00–15.00	3.00	[3.28–5.43]	a	A
	T2	4.33 ± 2.27	3.00–16.00	3.00	[3.27–5.39]	a	A
	T3	4.28 ± 2.23	3.00–16.00	3.00	[3.23–5.32]	a	A
Test (*n* = 20)	T0	4.16 ± 1.84	3.00–15.00	3.00	[3.30–5.02]	a	A
	T1	3.90 ± 1.58	3.00–15.00	3.00	[3.16–4.64]	a,b	A
	T2	3.78 ± 1.48	3.00–14.00	3.00	[3.09–4.48]	b	A
	T3	3.73 ± 1.39	3.00–12.00	3.00	[3.08–4.37]	b	A
Patient‐level							

Control (*n* = 20)	T0	4.46 ± 1.05	3.40–8.03	4.23	[3.97–4.95]	a	A
	T1	4.33 ± 1.09	3.27–8.11	4.09	[3.82–4.85]	a,b	A
	T2	4.32 ± 1.07	3.40–8.00	4.06	[3.82–4.82]	a	A
	T3	4.26 ± 1.08	3.29–8.00	4.05	[3.76–4.77]	b	A
Test (*n* = 20)	T0	4.19 ± 0.73	3.46–6.33	4.06	[3.85–4.54]	a	A
	T1	3.92 ± 0.67	3.29–5.80	3.76	[3.61–4.24]	a,b	A
	T2	3.81 ± 0.67	3.17–5.67	3.59	[3.49–4.12]	b	A
	T3	3.74 ± 0.60	3.13–5.57	3.54	[3.46–4.02]	b	A

Abbreviations: max, maximum; min, minimum; SD, standard deviation; T0, baseline; T1, 1 month; T2, 3 months; T3, 6 months.

^a^ Means presenting at least one identical letter are not significantly different (*p* > 0.05). Intragroup analysis considered all the time frames of the study for the comparisons (lowercase letters), while intergroup analysis considered only Control vs. Test comparison at each time frame (uppercase letters).

For VAS, baseline values were comparable between groups: 6.80 ± 2.19 (95% CI: 5.77–7.83) in the control group and 7.15 ± 2.28 (95% CI: 6.08–8.22) in the test group. Over time, both groups exhibited a slight increase in mean values. At T3, the control group reported a mean of 9.35 ± 0.88 (95% CI: 8.94–9.76), while the test group recorded 8.45 ± 1.57 (95% CI: 7.71–9.19). Intragroup analysis showed no statistically significant changes over time in either group. Intergroup comparisons also revealed no statistically significant differences between the test and control groups at any time point. Detailed descriptive and comparative data are reported in Table 9.

**TABLE 9:** Descriptive statistics and intragroup/intergroup comparisons of VAS values across all time points (T0–T3) in the Test and Control groups.

Group	Time	Mean ± SD	Range	Median	CI 95%	Intragroup (T0–T3)^a^	Intergroup (control‐test)^a^
Control (*n* = 20)	T0	6.80 ± 2.19	3.00–10.00	6.00	[5.77–7.83]	a	A
	T1	7.35 ± 1.84	4.00–10.00	7.00	[6.49–8.21]	a	A
	T2	7.05 ± 2.09	3.00–10.00	7.00	[6.07–8.03]	a	A
	T3	9.35 ± 0.88	7.00–10.00	10.00	[8.94–9.76]	a	A
Test (*n* = 20)	T0	7.15 ± 2.28	2.00–10.00	8.00	[6.08–8.22]	a	A
	T1	8.10 ± 1.77	3.00–10.00	8.00	[7.27–8.93]	a	A
	T2	7.35 ± 2.41	1.00–10.00	7.50	[6.22–8.48]	a	A
	T3	8.45 ± 1.57	4.00–10.00	9.00	[7.71–9.19]	a	A

Abbreviations: max, maximum; min, minimum; SD, standard deviation; T0, baseline; T1, 1 month; T2, 3 months; T3, 6 months.

^a^ Means presenting at least one identical letter are not significantly different (*p* > 0.05). Intragroup analysis considered all the time frames of the study for the comparisons (lowercase letters), while intergroup analysis considered only Control vs. Test comparison at each time frame (uppercase letters).

## 4 Discussion

This single‐blind RCT evaluated the adjunctive efficacy of a hyaluronic acid (HA)‐based gel compared with 0.20% chlorhexidine (CHX) in periodontal patients over a 6‐month follow‐up. The study design allowed for a comprehensive analysis of both tooth‐ and patient‐level outcomes, encompassing clinical periodontal parameters, tissue‐related indices, and patient‐reported satisfaction.

The first null hypothesis, which assumed no significant differences in periodontal outcomes between the HA‐based gel and CHX, was not rejected, as intergroup comparisons did not reveal statistically significant differences for the vast majority of clinical parameters at any time point. The only exception was represented by BoP at the tooth‐level, where limited intergroup differences were observed at specific time points, although their magnitude was modest and not consistently confirmed at the patient‐level. Conversely, the second null hypothesis, which postulated no differences in patient satisfaction, was accepted, since VAS scores did not significantly differ between groups at any stage of follow‐up. Overall, both treatment protocols resulted in improvements in several clinical parameters over time, confirming the effectiveness of adjunctive therapies in nonsurgical periodontal treatment. It should also be noted that the magnitude of the observed changes was relatively small, particularly for parameters such as PPD and CAL, where mean differences were in the range of a few tenths of a millimeter. Such variations may fall within the limits of examiner measurement variability and should therefore be interpreted with caution. In this context, the distinction between statistical significance and clinical relevance becomes particularly important, as statistically detectable changes do not necessarily translate into meaningful clinical benefits.

These findings are in line with the current literature supporting HA as an adjunct to nonsurgical periodontal therapy. Early randomized studies showed that HA application following scaling and root planing (SRP) could enhance PD reduction and reduce bacterial recolonization (Eick et al. 2013; Pilloni et al. 2023; Al‐Abbadi et al. 2025; Gundogdu Ezer and Gunpinar 2025). More recent systematic reviews and meta‐analyses have suggested that HA may improve inflammatory parameters and clinical attachment levels, although the magnitude of these effects varies depending on formulation, application protocol, and follow‐up duration (Eliezer et al. 2019; Alshehri and Alharbi 2023; Aldahlawi 2023).

Two recent randomized trials are particularly relevant in this context. Bertl et al. (2024) evaluated repeated HA application during supportive periodontal care and reported a reduction in sites requiring re‐instrumentation, together with a trend toward improved pocket closure over 12 months. Similarly, Benyei et al. (2024) investigated the combined use of sodium hypochlorite/amino acids and cross‐linked HA in persistent pockets, reporting improved clinical outcomes compared with instrumentation alone.

Unlike most previous studies, which compared HA with SRP alone, the present trial directly compared HA with CHX. This head‐to‐head design is clinically relevant, as CHX remains the most commonly used adjunct for plaque control. While effective in the short term, CHX is associated with well‐known side effects, including tooth staining and taste alteration (Poppolo Deus and Ouanounou 2022; James et al. 2017; Sun et al. 2024). Within this context, the present results suggest that HA can achieve clinical outcomes comparable to CHX on inflammation‐related parameters, while maintaining a favorable safety and tolerability profile, in agreement with previous reviews and clinical studies (Eliezer et al. 2019; Alshehri and Alharbi 2023; Graziani et al. 2024; Bhati et al. 2022). In recent years, increasing attention has also been directed toward natural products as potential alternatives to chlorhexidine. For example, extra‐virgin olive oil has been investigated in clinical settings for the management of plaque‐induced gingival inflammation. A recent clinical study demonstrated that daily rinsing with olive oil rich in polyphenols significantly reduced plaque accumulation and gingival inflammation over a 30‐day period, suggesting a potential antimicrobial and anti‐inflammatory effect associated with its bioactive phenolic compounds. These findings support the broader concept that natural bioactive substances may contribute to biofilm control while minimizing the adverse effects typically associated with long‐term chlorhexidine use (Zumbo et al. 2023).

With regard to specific clinical parameters, GR and TM remained stable in both groups throughout the study, which is consistent with the limited impact of nonsurgical adjunctive therapies on these variables (Eliezer et al. 2019). CAL improvements were observed over time in both groups, with slightly earlier changes in the HA group, although without significant differences between treatments. A similar consideration applies to BoP: although some differences were detected at specific time points at the tooth‐level, they were not confirmed at the patient‐level and appear to have limited clinical relevance. This interpretation is in line with previous reports showing that HA may support improvements in bleeding control and attachment gain, although with heterogeneous effect sizes across studies (Alshehri and Alharbi 2023; Olszewska‐Czyz et al. 2021).

It is also worth noting that combination approaches coupling chemomechanical biofilm disruption with cross‐linked HA may yield additive benefits. Ramanauskaite et al. (2023) demonstrated that sodium hypochlorite/amino acids plus xHyA adjunctive to debridement improved clinical outcomes over 6 months in a multicenter RCT. Although our trial compared HA directly with CHX rather than testing a combination protocol, the present findings support the view that HA‐based strategies may support periodontal improvement without introducing the adverse effects commonly associated with prolonged CHX use.

Patient‐reported outcomes confirmed that both treatments were well tolerated. The absence of differences in VAS scores suggests that, when adequate nonsurgical therapy and oral hygiene instruction are provided, patient satisfaction is high regardless of the adjunctive agent used. This is also consistent with the limited literature on patient‐reported outcomes associated with HA, which generally report good tolerance but not necessarily superior perceived benefit (Olszewska‐Czyz et al. 2021).

From a clinical standpoint, both HA and CHX can be considered effective adjuncts to nonsurgical periodontal therapy, providing comparable improvements in periodontal parameters. The choice between the two may therefore depend on individual patient factors, including tolerance, preference, compliance, and the potential for side effects.

The strengths of this study include its randomized design, the absence of drop‐outs, and the standardized treatment protocol applied across both groups. The direct comparison between HA and CHX adds clinically relevant information to the current literature.

The present trial has several limitations that should be acknowledged. The follow‐up period was restricted to 6 months, preventing any assessment of the long‐term stability of the observed improvements or potential late adverse effects. Only systemically healthy, nonsmoking individuals were included, which may not reflect the heterogeneous clinical populations encountered in daily practice, such as patients with systemic conditions (e.g., diabetes, cardiovascular disease) or at higher risk of disease progression. In addition, the trial did not include a placebo group or a “SRP‐only” arm, thereby precluding evaluation of the absolute adjunctive effect of either HA or CHX compared with mechanical therapy alone. The single‐blind nature of the trial could have introduced performance bias given the different formulations of the tested products (gel versus mouthwash). Moreover, the relatively small sample size and single‐center setting may have introduced a risk of type II error for some secondary outcomes and limit the external validity of our findings.

Future studies should include larger and more diverse populations, longer follow‐up periods, and standardized protocols for HA application. The inclusion of microbiological and biomarker analyses may help clarify the mechanisms underlying the observed clinical effects, while cost‐effectiveness evaluations could further support clinical decision‐making.

## 5 Conclusions

Within the limitations of this RCT, adjunctive use of an HA gel provided slightly better but comparable outcomes in PPD, PCR, BoP, and CAL compared with 0.20% CHX over 6 months. GR and TM remained unchanged, consistent with previous evidence that nonsurgical adjuncts rarely influence these parameters. Both protocols were well tolerated, with no AEs reported and comparable patient satisfaction. These findings suggest that HA may represent a viable alternative to CHX for the adjunctive treatment of periodontal patients.

## Author Contributions

**Andrea Scribante:** conceptualization, data curation, supervision, formal analysis, methodology, project administration, validation, visualization, writing – review and editing. **Matteo Pellegrini:** data curation, methodology, project administration, resources, software, supervision, validation, visualization, writing – original draft. **Andrea Butera:** conceptualization, data curation, methodology, project administration, supervision, validation, visualization, writing – review and editing. **Maurizio Pascadopoli:** formal analysis. **Karyna Chekal:** validation, visualization, data curation, investigation.

## Funding

The authors have nothing to report.

## Ethics Statement

The study was conducted in accordance with the ethical standards outlined in the Declaration of Helsinki. Approval was granted by the Internal Review Board of the institution (Protocol number 2022‐0302; date of approval: March 3, 2022), and the trial was prospectively recorded on ClinicalTrials.gov under the identifier NCT05312606.

## Consent

Each participant enrolled in the study provided written informed consent prior to inclusion.

## Conflicts of Interest

The authors declare no conflicts of interest.

## Supporting information


**Table S1:** CONSORT 2025 checklist.

## Data Availability

Data produced and examined within this investigation can be obtained by contacting the corresponding authors, provided the request is justified and appropriate.
